# Dental traumatic avulsions: an analysis of the performance and maintenance of replanted teeth in the first-year post-injury

**DOI:** 10.21142/2523-2754-1302-2025-238

**Published:** 2025-05-16

**Authors:** Bhárbara Marinho Barcellos, Melissa Feres Damián, Talita Freitas da Silva, Marcos Augusto Lourenco da Silva, Leticia Kirst Post, Josué Martos, Cristina Braga Xavier

**Affiliations:** 1 Hospital for Rehabilitation of Craniofacial Anomalies, University of Sao Paulo. Bauru, Brazil. Universidade de São Paulo Hospital for Rehabilitation of Craniofacial Anomalies University of Sao Paulo Bauru Brazil; 2 Department of Semiology and Clinic, School of Dentistry, Federal University of Pelotas. Pelotas, Brazil. melissaferesdamian@gmail.com, josue.sul@terra.com.br Universidade Federal de Pelotas Department of Semiology and Clinic School of Dentistry Federal University of Pelotas Pelotas Brazil melissaferesdamian@gmail.com josue.sul@terra.com.br; 3 Federal University of Pelotas. Pelotas, Brazil tatah.fds@gmail.com, gutto_gutto@hotmail.com Universidade Federal de Pelotas Federal University of Pelotas Pelotas Brazil tatah.fds@gmail.com gutto_gutto@hotmail.com; 4 Department of Oral and Maxillofacial Surgery, School of Dentistry, Federal University of Pelotas. Pelotas, Brazil. letipel@hotmail.com, cristinabxavier@gmail.com Universidade Federal de Pelotas Department of Oral and Maxillofacial Surgery School of Dentistry Federal University of Pelotas Pelotas Brazil letipel@hotmail.com cristinabxavier@gmail.com

**Keywords:** tooth avulsion, dental replantation, dental trauma, avulsión dentaria, reimplantación dental, traumatismos dentales

## Abstract

**Aim:**

This study aims to examine the factors influencing the decision to perform replantation following avulsion of permanent teeth, as well as factors affecting the maintenance of replanted teeth in the first-year post-injury.

**Material and Methods:**

This retrospective analysis involved examining dental records and periapical radiographs of patients with avulsed permanent teeth. Demographic information, details regarding traumatic lesions, and treatment specifications were extracted from dental records. Radiographs obtained during the initial appointment were used to assess root maturity, and the ones at the one-year follow-up examination evaluated the maintenance of replanted teeth, along with the presence of endodontic therapy, bone loss, periapical lesions, and inflammatory or replacement resorption. Chi-square tests and hierarchical Logistic Regression Analysis were employed to identify factors associated with replantation success and maintenance of replanted teeth after one year.

**Results:**

Data from 106 patients and 154 avulsions were collected according to predefined criteria. Of the 154 teeth, 102 (76.2%) underwent replantation. Patients over 13 years of age at the moment of the traumatic injury, those with avulsion of more than one tooth, and those with associated alveolar bone fractures were less likely to undergo replantation. In the first-year post-injury, 71 of the 102 replanted teeth (70.3%) were retained. Tooth loss within the first-year follow-up was more likely in male patients, individuals over 13 years old at the moment of the avulsion, and those with associated alveolar bone fractures. **Conclusions:** Patient age and alveolar bone fractures during traumatic injuries were identified as factors associated with both replantation outcomes. Patient gender influenced the presence of the tooth in the first-year post-injury, while avulsion of more than one tooth showed an association with replantation performance.

## RESUMEN

**Objetivo:**

Este estudio tiene como objetivo examinar los factores que influyen en la decisión de realizar una reimplantación después de la avulsión de los dientes permanentes, así como los factores que afectan el mantenimiento de los dientes reimplantados en el primer año después de la lesión.

**Material y métodos:**

Este análisis retrospectivo implicó examinar registros dentales y radiografías periapicales de pacientes con dientes permanentes avulsionados. La información demográfica, los detalles sobre lesiones traumáticas y las especificaciones de tratamiento se extrajeron de los registros dentales. Las radiografías obtenidas durante la cita inicial se utilizaron para evaluar la madurez radicular, y las del examen de seguimiento al año evaluaron el mantenimiento de los dientes reimplantados, junto con la presencia de terapia de endodoncia, pérdida ósea, lesiones periapicales e inflamación o reemplazo. Se emplearon pruebas de chi-cuadrado y análisis de regresión logística jerárquica para identificar factores asociados con el éxito de la reimplantación y el mantenimiento de los dientes reimplantados después de un año.

**Resultados:**

Se recogieron datos de 106 pacientes y 154 avulsiones según criterios predefinidos. De los 154 dientes, 102 (76,2%) fueron reimplantados. Los pacientes mayores de 13 años en el momento de la lesión traumática, aquellos con avulsión de más de un diente y aquellos con fracturas de hueso alveolar asociadas tenían menos probabilidades de someterse a un reimplante. En el primer año posterior a la lesión, se conservaron 71 de los 102 dientes reimplantados (70,3%). La pérdida de dientes durante el primer año de seguimiento fue más probable en pacientes masculinos, personas mayores de 13 años en el momento de la avulsión y aquellos con fracturas de hueso alveolar asociadas.

**Conclusiones:**

La edad del paciente y las fracturas del hueso alveolar durante lesiones traumáticas se identificaron como factores asociados con ambos resultados del reimplante. El sexo del paciente influyó en la presencia del diente en el primer año posterior a la lesión, mientras que la avulsión de más de un diente mostró una asociación con el rendimiento de la reimplantación.

## INTRODUCTION

Traumatic dental injuries (TDI) are recognized as a significant public health concern^1^. Avulsion, defined as the complete displacement of the tooth from its socket^2^, represents one of the most serious TDIs, causing severe damage to the supporting tissues as well as the vascular and nervous structures^3^.

Following epidemiological investigations, avulsion in the permanent dentition constitutes 0.5-16% of traumatic lesions, exhibiting a higher prevalence among males, particularly in children and adolescents aged 7-12 years^4^. This heightened occurrence is attributed to various factors, notably recreational activities, interpersonal violence, and automobile accidents^2^^,^^5^^-^^8^. The maxillary central incisors frequently bear the brunt of such injuries^2^^,^^7^. Consequently, the oral health-related quality of life for individuals experiencing avulsion may be significantly impacted^9^.

The recommended protocol for tooth avulsion involves immediate replantation at the site of the accident, conducted promptly after the traumatic injury^3^. When immediate replantation is not feasible, the avulsed teeth should be preserved in a suitable medium until timely replantation can be performed to mitigate any adverse impact on prognosis^10^. Furthermore, aspects such as the choice and duration of splinting, considerations related to endodontic procedures (aligned with root maturity), and other relevant factors must be meticulously addressed in the management of avulsed teeth^3^. Deviating from established treatment recommendations can compromise the outcomes and survival rates of replanted teeth, influencing patients' quality of life^11^^,^^12^. This deviation not only affects clinical outcomes but also contributes to an escalation in the direct costs associated with treatment^4^. 

Recognizing the importance of precise replantation treatment for avulsed permanent teeth, this study aimed to analyze factors associated with the decision to either undertake or abstain from replantation following avulsions. Additionally, the investigation sought to explore factors influencing the maintenance of replanted teeth during their first-year post-injury.

## MATERIAL AND METHODS

### Study design and ethical aspects

This observational retrospective study was approved by an Institutional Research Ethics Committee (CAAE: 77687417.2.0000.5318). Its report was made according to STROBE guidelines ^(^^13^.

### Sample

The study sample comprised dental records from patients with avulsed permanent teeth treated at a specialized dentoalveolar trauma reference service in southern Brazil from January 2002 to September 2018. Considering the specific focus on a 1-year follow-up after replantation and the emergence of the COVID-19 pandemic, patient and avulsion data collection was concluded in September 2018, allowing for assessment in September 2019. The decision to limit data collection to this timeframe was influenced by both the designated follow-up period and the dynamic circumstances introduced by the pandemic. Additionally, justifying data collection until 2019 is the alteration in International Association of Dental Traumatology (IADT) treatment protocols starting in 2020^3^. This precaution was taken to ensure the coherence and comparability of results, recognizing the potential impact of protocol changes on the outcomes under investigation.

The eligibility criteria encompassed: (a) patients experiencing permanent tooth avulsion; (b) individuals with a minimum one-year follow-up; and (c) dental records containing comprehensive data, complemented by high-quality radiographs suitable for accurate interpretation. Conversely, exclusion criteria were applied to (a) patients presenting with alternative forms of dentoalveolar trauma; (b) cases involving avulsion exclusively in deciduous teeth; (c) instances where the initial and 1-year follow-up radiographs were unavailable; and (d) radiographs lacking the requisite quality for precise interpretation.

### Data collection

Data extracted from dental records comprised: (a) patient’s demographic information, specifically gender and age at the moment of the avulsion; (b) comprehensive details regarding the traumatic lesion, including specifics about the avulsed tooth, the number of avulsed teeth, the etiology of the trauma, the location of the injury, as well as any accompanying soft tissue injuries or alveolar bone fractures; and (c) information about the treatment administered, such as the duration the tooth was out of the oral cavity, the storage medium utilized, and the type of splint employed, along with the duration for which it was maintained.

Periapical radiographic examinations also were used to collect data. The initial exam, conducted during the first appointment, scrutinized root maturity, discerning between an open or closed apex. Subsequently, an evaluation of records obtained during the 1-year follow-up encompassed an assessment of replanted tooth maintenance (yes or no) and the presence of diverse outcomes, including endodontic therapy, bone loss, periapical lesions, inflammatory resorption or replacement resorption (yes or no) in the replanted teeth. This analysis was achieved within a controlled environment utilizing a view box device (LED View Box, Biotron Equipamentos Médicos, Santa Rita do Sapucaí, Minas Gerais, Brazil) and a 4x magnifying glass. All data acquisition was executed by two evaluators and the kappa test was used to measure the degree of agreement between the evaluators.

### Statistical analysis

The entirety of the data underwent tabulation in duplicate to mitigate potential sources of bias, utilizing a Microsoft Excel® spreadsheet (Microsoft Corporation, Redmond, USA). Initially, a comprehensive descriptive statistics analysis was conducted to assess the data related to avulsions and replanted teeth. Subsequently, a chi-square test with a 5% significance level was employed to scrutinize factors associated with tooth replantation performance and the maintenance of replanted teeth within the first-year post-injury. To discern possible confounding factors, independent variables exhibiting a statistical association in the chi-square test were subjected to a hierarchical Logistic Regression Analysis. The execution of all statistical tests was carried out using the IBM™ SPSS Statistics software (International Business Machines Corporation, Armonk, New York, United States).

## RESULTS

The kappa test was used to measure interobserver agreement. The result was a value of 0.77 with a moderate agreement based on the Landis and Koch ’s criteria.

### Patients and Avulsion Epidemiological Data

Throughout the investigated period, 827 individuals sought treatment at the reference service, with 195 (23.5%) experiencing avulsion incidents. Through the application of the predefined inclusion and exclusion criteria, data from 106 patients (54.3% of the 195 initially registered) were analyzed, yielding insights into a total of 154 avulsed teeth. Exclusion primarily resulted from insufficient patient follow-up duration (less than 1 year) and the absence or inadequate quality of radiographic exams.

The prevalence of avulsion was higher in male patients (66%), predominantly affecting the upper central incisors (82.1%) and a younger demographic, with a mean age of 17.52 (±11.8) years at the moment of the avulsion injury (ranging from 6 to 60 years, and a median age of 13 years). While the majority of patients experienced single-tooth avulsion incidents (69.8%), a noteworthy subset exhibited 2, 3, or even 6 avulsed teeth. Falls and sports activities, including cycling accidents, emerged as primary etiological factors, accounting for 33% and 27.4%, respectively. The majority of traumatic incidents occurred in public areas such as streets (64.8%) or at home (23.8%). Soft tissue injuries and alveolar bone fractures were present in 68.7% and 30.5% of avulsion cases, respectively (Table 1).

**Table 1:** Epidemiological data of 102 replants performed after avulsion of 154 teeth in a southern Brazil reference service

Variable	Category	Absolute Value	Relative Value
Tooth Group	Upper Central Incisor	86	84.3%
	Upper Lateral Incisor	11	10.8%
	Lower Central Incisor	3	2.9%
	Lower Lateral Incisor	1	1.0%
	Lower Premolar	1	1.0%
Number of Avulsed Teet	1 Tooth	60	58.8%
	>1 Tooth	42	41.2%
Storage Medium*	Dry	51	52.6%
	Milk	12	12.4%
	Saline	13	13.4%
	Saliva	9	9.3%
	Others	12	14.4%
Splint*	Rigid	45	51.4%
	Soft	37	42.04%
	Non-splint	6	6.82%
Splint Period*	≤2 weeks	5	6.25%
	>2 weeks	75	93.75%
Maturity of the Root	Open apex	30	29.4%
	Closed apex	72	70.6%

* Independent variable with missing data

### Replanted Teeth

A total of 102 out of the 154 avulsed teeth (76.2%) underwent replantation, with a predominant occurrence in upper central incisors (84.3%). Among the replanted teeth, 72 (70.6%) exhibited a closed apex, while 30 (29.4%) displayed an open apex. The replantations were conducted promptly, with 29.5% occurring within the first-hour post-avulsion, 41% between 1 and 24 hours, and 29.5% exceeding 24 hours after the traumatic injury. A majority of teeth were replanted under suboptimal conditions regarding the storage medium. Single-tooth avulsion was prevalent, accounting for 58.8% of the replantation cases (Table 1).

The application of the chi-square statistical test highlighted associations between patients' age at the moment of avulsion, the number of avulsed teeth, and alveolar bone fractures with the achievement of tooth replantation. This association was substantiated through logistic regression testing. Multivariate analysis further revealed that patients over 13 years old, those involved in multiple avulsions, and those with associated alveolar bone fractures had a heightened likelihood of not undergoing replantation after avulsion (Table 2).

**Table 2:** Odds ratio (OR) values in the hierarchical Logistic Regression Analysis to replant teeth

		OR	IC 95%	p value
Age*	≤13 years old	1		
	>13 years old	8.33	3.03 - 22.91	<0.001
Number of Avulsed Teeth	1 Tooth	1		
	>1 Tooth	3.06	1.30 - 7.19	0.010
Alveolar Bone Fracture	No	1		
	Yes	2.52	1.06 - 5.98	0.036

* Variable dichotomized by the median for statistical analysis

### 1-year follow-up

In the radiographic assessment conducted 1-year post-injury, despite encountering disturbances such as root resorption (inflammatory or replacement), bone loss, and periapical lesions (Table 3), 71 out of the 102 replanted teeth were present (70.3%). Both chi-square and logistic regression statistical analyses revealed significant associations between patients' gender and age, as well as the concurrent presence of alveolar bone fractures, with the maintenance or loss of the replanted tooth at the 1-year follow-up. The likelihood of tooth loss within the first year was notably higher for male patients, individuals over 13 years old at the moment of traumatic injury, and cases involving associated alveolar bone fractures, as indicated by the multivariate analysis (Table 4).

**Table 3:** Radiographic outcomes - 71 present teeth in the 1-year follow-up after replant (represented 70.3% of replanted teeth)

	Endodontic Therapy		Bone Loss		Apical Lesion		Inflammatory Resorption		Replacement Resorption	
	N	%	N	%	N	%	N	%	N	%
No	7	9.9	44	62	63	88.7	62	87.3	29	40.8
Yes	64	90.1	27	38	8	11.3	9	12.7	42	59.2
Total	71	100	71	100	71	100	71	100	71	100

**Table 4:** Odds ratio (OR) values in the hierarchical Logistic Regression Analysis to 1-year follow-up tooth loss

		OR	IC 95%	p value
Gender	Female	1		
	Male	6.02	2.29 - 15.79	<0.001
Age	≤13 years old	1		
	>13 years old	5.64	2.35 - 13.58	<0.001
Alveolar Bone Fractures	No	1		
	Yes	5.43	1.99 - 14.78	0.001

## DISCUSSION

This study, based on information derived from a reference service in the treatment of dentoalveolar trauma, outlined an assessment of factors correlated with the performance of replantation in avulsed teeth, as well as the subsequent maintenance of these teeth during first-year post-traumatic injury. Despite existing literature containing information regarding the maintenance and survival of replanted teeth after avulsion^14^^-^^16^, it is noteworthy that there is a deficiency of studies in databases that investigated the characteristics of patients who did not undergo replantation after tooth avulsion. The identified gap emphasizes the significant relevance of the data obtained in this study.

Out of the 154 teeth subjected to evaluation, 102 were replanted, accounting for a representation exceeding 75% of the examined sample (76.2%). The findings align with those reported in the study conducted by Wang (2019) ^(^^17^, wherein 18.8% (76 cases) of avulsed teeth were observed not to undergo replantation. The endorsed treatment for permanent tooth avulsion is replantation^3^, however, when the replants are performed under adverse conditions, replanted teeth may initiate a resorption process leading to eventual loss. Conversely, in cases where avulsed teeth are not replanted, patients may incur damages, as a reduction in their quality of life ^(^^9^and an associated increase in the overall treatment cost^4^. Hence, whenever feasible, the replantation of avulsed teeth is deemed crucial.

Another significant outcome of this study is the notably low rate of tooth loss recorded in the 12-month follow-up, with 70.3% of replanted teeth maintaining their presence and functionality. Supporting and reinforcing these findings, Wang et al. (2019) ^(^^17^reported a tooth loss rate of 69.7% within the first year following replantation in their study. This finding holds particular significance considering the challenging conditions under which a great number of replantations were performed. Consequently, the decision to proceed with treatment, even in adverse circumstances, stands out as a crucial clinical choice, supported by the evidence derived from this investigation. The endorsement of replantations, even in challenging scenarios, is reinforced by the considerable percentage (28.9%) of teeth exhibiting an intact root surface (functional healing) in the first-year follow-up. Functional healing represents the most desirable outcome post-replantation, and the authors recognize that this percentage may stabilize or decrease over time. This aligns with existing literature, such as studies reporting rates of 21.4% for functional healing after 4 years of follow-up^17^. Petrovic et al. (2010) ^(^^18^found that 68.7% of replanted teeth were still present, with 15% showing functional healing after 2.5 years of follow-up. Additionally, Muller et al. (2020) ^(^^19^observed rates of 24.5% for teeth with functional healing and 63% for teeth still present, with an average follow-up of 3.5 years. However, concerning tooth maintenance, a recent study indicated an overall survival rate of avulsed/replanted permanent teeth at 50% after 5.5 years^14^. It is conceivable that with an extended follow-up period in the present study, these percentages of remaining present teeth might experience a decline.

Regarding the outcomes following tooth avulsion and replantation, a notable correlation between patient age and the observed results was found. Specifically, the patient's age exhibits a discernible association with both the execution of replantations subsequent to avulsion and the maintenance of replanted teeth within the initial year post-traumatic injury. Notably, individuals surpassing 13 years of age at the time of avulsion manifest an elevated likelihood of forgoing tooth replantation and experiencing tooth loss within the initial year of follow-up, in contrast to counterparts under the age of 13. This finding aligns with Rhouma and McMahon's (2012) ^(^^20^, that emphasized the significant role of patient age in the maintenance and survival of replanted teeth post-avulsion.

The elucidation of such findings may be attributed to the meticulous monitoring protocols implemented within the investigated dental service, coupled with the comprehensive information dissemination to patients and their guardians. As underlined by Glendor (2000) ^(^^21^, the commitment and active involvement of the family unit play a fundamental role in shaping the temporal and financial dimensions of dental trauma treatment. Notably, insights from Némat (2023) ^(^^22^presumed that the information comprehension and the attendance to follow-up appointments is contingent upon the age and maturity level of the patient. Consequently, discussions concerning tooth prognosisand long-term treatment plans are recommended to be hold and enlightened during follow-up consultations, where guardians may possess an enhanced capacity to retain pertinent information. Given that patients within this age cohort are typically accompanied by parents or guardians, there exists a heightened frequency of appointments compared to their adolescent counterparts. This heightened frequency facilitates early detection of inflammatory resorptions or another changes, thereby enabling timely interventions conducive to the preservation of reimplanted teeth. 

It is noteworthy that Wang (2019) ^(^^17^and Coste (2020) ^(^^14^, have reported elevated survival rates of reimplanted teeth in patients exceeding 16 years of age, attributing these outcomes to rhizogenesis. However, the standpoint presented by Bastos et al. (2014) ^(^^23^emphasizes the necessity of age as a factor in the loss of avulsed teeth independently from considerations of root maturity. So, regarding the identification of age as a predictive factor for replantation execution, no other study exploring this specific association could be identified. However, it is hypothesized that additional factors such as the presence of alveolar fractures and the quantity of avulsed teeth, might have played a role in influencing the observed lower probability of replantation procedures.

The alveolar bone fracture associated with avulsion also represented a factor that influenced both evaluated outcomes. When avulsion coincides with alveolar fracture, it emerges as the primary cause of tooth loss, posing a challenge to the healing process^24^. The prognosis of tooth loss following complex dentoalveolar trauma hinges on the clinical features of the fracture, with particular emphasis on the status of the alveolar socket. The use of a longer splinting time as a treatment modality also plays a fundamental aspect in these results. The likelihood of tooth loss in cases with a damaged alveolar socket was 97.2% (95% CI, 85.4-99.9), contrasting with a 22% probability (95% CI, 2.8-60) in cases with an intact socket^25^.

Notably, alveolar bone fractures manifest more frequently in young male adults, with the segment commonly involving two teeth^26^. While this study did not specifically assess the relationship of alveolar bone fracture with other factors, the presence of both alveolar bone fracture and a higher number of avulsed teeth were identified as factors diminishing the chances of successful replantation. Concurrently, the presence of alveolar bone fracture and male gender emerged as predictors for a likelihood of dental loss in the first year of follow-up.

The avulsion of multiple teeth also exhibited a diminished likelihood of having their teeth replanted. In a Brazilian study conducted in a specialized service for dentoalveolar trauma treatment, with avulsion being the predominant injury, the engagement of a greater number of teeth (3 or more elements) was statistically linked to an escalation in the severity of the traumatic injury^27^. More severe traumas may pose challenges to treatment, thereby diminishing the probability of successfully replanting avulsed teeth.

Avulsion affected more men (66%) than women (34%), which is consistent with several reports focused on dental avulsion and traumatic dental injuries epidemiology. ^(^^27^^-^^29^, which outnumbered female patients by a factor of 1.65^30^. This epidemiological data holds significance, as the study indicates that male gender exerted a negative influence on the retention of replanted teeth during their initial year of follow-up.

One of the limitations of this study was that 71 patients (36.4%) did not respect the periodic follow-up. Perhaps, there is a lack of information about this subject, and the population should be encouraged through government educational and preventive campaigns, to deepen their knowledge about what to do in case of dental trauma. An additional limitation pertains to the duration of follow-up examined in this study, confined to the first-year. As previously underscored, the rates of maintenance and survival for replanted teeth exhibit a diminishing trend with extended follow-up periods^14^.

In summary, over the course of 16 years within this dental service, 23.5% of patients suffered avulsion of permanent teeth, being male and younger patients the most prevalent. Upper central incisors were most affected teeth and sports activities, including cycling, were the main etiologies. Although the concomitant alveolar bone fracture has been not prevalent, this factor showed association both with the possibility of performance of the replant after the avulsion and the maintenance of the replanted tooth after 1-year. Additionally, the patients' age demonstrated an association with both analyzed outcomes and it was observed that the female gender exhibited an association with the retention of the tooth during the first-year follow-up period. Another finding worthy of note is that the occurrence of multiple tooth avulsions may influence the decision or viability of performing replantations. Further investigation into this variable could provide valuable information about the complexities of managing cases involving multiple avulsed teeth.

## CONCLUSIONS

Factors such as patient age and the occurrence of alveolar bone fractures during traumatic injuries were identified as influencing replantation outcomes. Additionally, the patient's gender affected tooth retention during the first year following the injury. Furthermore, the avulsion of multiple teeth was linked to variations in replantation performance.
